# Clinical and imaging characteristics of patients with axial psoriatic arthritis: Baseline findings from the GESPIC-axial-PsA cohort

**DOI:** 10.1177/1759720X261472985

**Published:** 2026-09-03

**Authors:** Henriette Baum, Murat Torgutalp, Valeria Rios Rodriguez, Judith Rademacher, Mikhail Protopopov, Hildrun Haibel, Burkhard Muche, Kay-Geert Hermann, Denis Poddubnyy, Fabian Proft

**Affiliations:** 1 Department of Gastroenterology, Infectiology and Rheumatology (including Nutrition Medicine), 14903 Charité – Universitätsmedizin Berlin, Corporate Member of Freie Universität Berlin and Humboldt-Universität zu Berlin, Berlin, Germany; 2 Department of Rheumatology and Clinical Immunology, 14903 Charité - Universitätsmedizin Berlin, Corporate Member of Freie Universität Berlin and Humboldt-Universität zu Berlin, Berlin, Germany; 3 Fraunhofer Institute for Translational Medicine and Pharmacology (ITMP), Berlin, Germany; 4 Department of Radiology (CCM), 14903 Charité - Universitätsmedizin Berlin, Campus Mitte, Humboldt-Universität Zu Berlin, Freie Universität Berlin, Berlin, Germany; 5 Division of Rheumatology, University of Toronto and Schroeder Arthritis Institute, University Health Network, Toronto, ON, Canada

**Keywords:** psoriatic arthritis, spondyloarthritis, axial psoriatic arthrtis, imaging, observational study

## Abstract

**Background:**

Axial disease is a domain of psoriatic arthritis (PsA), referred to as axial-PsA. Despite recent advances and increasing recognition, axial-PsA remains poorly defined; validated classification criteria and longitudinal cohorts are lacking.

**Objectives:**

We aimed to characterise axial-PsA and delineate phenotypes in an imaging-confirmed cohort.

**Design:**

GESPIC-axPsA, a dedicated arm of the German Spondyloarthritis Inception Cohort (GESPIC), is a prospective, monocentric, study including patients with psoriasis and clinically diagnosed axial-PsA confirmed by imaging. Baseline assessments included clinical phenotyping, laboratory testing, biosample-collection, radiographs of the axial skeleton, and MRI of the whole spine and sacroiliac joints (SIJs).

**Methods:**

The analysis was primarily descriptive, characterizing baseline clinical and imaging features of patients with imaging-confirmed axial-PsA; categorical and continuous variables were summarized using appropriate measures based on their distribution, and subgroup comparisons were performed using chi-square or Fisher’s exact tests for categorical variables and Mann–Whitney U tests for continuous variables after assessing normality.

**Results:**

We enrolled 107 axial-PsA patients (55% female; mean age 44.8 years); 78.5% had active psoriasis, 78.6% had inflammatory back pain (IBP), and 48.6% were HLA-B27 positive. Radiographic sacroiliitis was present in 45.7%. On MRI, active/structural SIJ lesions were seen in 53.3%/73.3%; spinal active/structural lesions in 60.0%/53.3%. Both Classification criteria for axSpA (ASAS) and PsA (CASPAR) were simultaneously fulfilled in 60.7%. We identified 20 patients (18.7%) with spine-only involvement—spinal MRI lesions without SIJ lesions on radiography or MRI. Compared with the remainder, the spine-only group was older, more often female, less frequently HLA-B27–positive, and had back pain less consistent with IBP (all p<0.05).

**Conclusions:**

In our imaging-confirmed axial-PsA cohort using standardized whole-spine and SIJ MRI, we observed marked phenotypic heterogeneity. Approximately one fifth had isolated spinal involvement; this subgroup differed by age, sex, HLA-B27, and IBP features, with imaging distributions distinct from axSpA—supporting the need for disease-specific classification criteria.

## Introduction

Psoriatic disease^1,2^ describes a systemic inflammatory disorder with potential multi-organ involvement, most commonly referring to the co-occurrence of psoriasis (PsO) and psoriatic arthritis (PsA). PsO is a chronic, inflammatory multisystemic skin disease affecting over 60 million people worldwide.^3–5^ PsA is a progressive and potentially debilitating disease characterized by a heterogeneous clinical presentation encompassing five primary domains: peripheral arthritis, enthesitis, dactylitis, axial involvement as well as skin and nail involvement, which all may occur individually or in combination.^6–8^ PsA shows no consistent sex predilection, with broadly similar incidence in men and women, and typically manifests in the fourth to fifth decade.^9^ Management is complicated by clinical heterogeneity, requiring domain-specific approaches.^10^

Axial involvement in psoriatic arthritis (axial-PsA) was first described by Moll and Wright in 1973, who highlighted the lack of a standardized definition.^11^ Reported prevalence estimates vary widely (e.g. 25-70%) depending on the criteria applied, and diagnosis remains inconsistent due to the absence of standardized and validated classification or operational criteria.^7,9,12–14^ PsA and axial-PsA are subsumed under the umbrella term of spondyloarthritis (SpA), which also includes axial spondyloarthritis (axSpA), and related disorders with both axial and peripheral manifestations.^15^ Compared to axSpA, axial-PsA is less frequently linked to inflammatory back pain and may be asymptomatic.^16^ Genetically, HLA-B27 is less prevalent in axial-PsA (14 - 44%) than in axSpA.^5,17^ A current hypothesis states that axial-PsA is predominantly characterized by ligament-centered soft tissue involvement, in contrast to the bone-centered pathology typical of axSpA.^18^ Whereas HLA-B27–associated bone marrow inflammation and osteitis are hallmarks of axSpA, axial-PsA primarily affects the spinal ligaments and entheses (“*ligamentitis*”). The proposed “*tree trunk and root*” model describes axial-PsA as exhibiting pronounced soft tissue ossification and ligament involvement (tree trunk) rather than the early entheseal inflammation (tree roots) characteristic of axSpA.^19,20^ Conventional radiography primarily identifies chronic structural lesions such as sacroiliitis, which are present in approximately 25–50% of patients with axial-PsA, as well as spinal changes including syndesmophytes and ankylosis.^21^ MRI not only visualizes active inflammation and thereby enables earlier diagnosis but can also detect certain structural lesions.^22^ While in axial SpA inflammation typically begins in the sacroiliac joints and only later extends to the spine—leading to diagnostic algorithms that prioritize SIJ imaging before spine imaging^23^—axial-PsA may present with isolated spinal involvement in a substantial proportion of patients.^24^ Therefore, spinal imaging should be incorporated into diagnostic evaluations of axial-PsA from the outset, ideally combining conventional radiography for chronic structural changes with MRI to capture active inflammatory (and potentially early) lesions, thus providing a more comprehensive clinical picture.^8,22,24^

Despite growing awareness of axial-PsA, key gaps remain.

i) The absence of a consensus definition or validated classification criteria results in heterogeneity across studies and uncertainty regarding prevalence, phenotype and disease course. Against this environment, the joint Axial Involvement in Psoriatic Arthritis (AXIS) project by the Assessment in SpondyloArthritis international Society (ASAS) and the Group for Research and Assessment of Psoriasis and Psoriatic Arthritis (GRAPPA) recently conducted a structured cross-sectional evaluation of axial involvement in PsA using standardised imaging.^25^
ii) Most data on treatment effects arise from post hoc analyses or SpA-focused studies, often without standardized imaging and dedicated assessment of symptoms specific for axial-PsA.^26–29^
iii) Furthermore, the relative scarcity of prospective, longitudinal cohorts’ limits biomarker discovery, understanding of disease mechanisms and progression, optimization of targeted therapies for this under-characterized disease domain, and robust evaluation of long-term structural damage.

In conclusion, clinical and genetic distinctions between axial-PsA and axSpA thus remain incompletely characterized, and current imaging criteria may overlook features relevant to axial-PsA.

To address these gaps, a new arm of the German Spondyloarthritis Inception Cohort focusing specifically on axial-PsA *(GESPIC-axPsA)* was initiated as a prospective study specifically designed to investigate patients with a clinically and imaging-confirmed diagnosis of axial-PsA. The protocol provides standardized longitudinal data and includes comprehensive data collection, such as clinical assessments, whole-spine and sacroiliac joint (SIJ) MRI plus radiography with central reading and biomarkers This approach enables detailed phenotypic and pathophysiological characterization, with the aim of advancing understanding in an area with limited dedicated research.

The primary objectives were to (1) observe and define the clinical and imaging characteristics of a well-characterized axial-PsA population and (2) to investigate disease trajectories and morphological patterns over time.

This article reports the baseline findings (cross-sectional) from this cohort.

## Methods

### Study design and participants

GESPIC is an ongoing, prospective study investigating the course and long-term outcomes of SpA.^30,31^ The GESPIC-axPsA arm is a prospective, monocentric, longitudinal observational study. Patients with a clinical diagnosis of axial-PsA were included if prior imaging supported the diagnosis and confirmation was reached through interdisciplinary consensus between rheumatologists and a radiologist with expertise in musculoskeletal imaging. If discordant opinions persisted following discussion, the patient was not enrolled in the study.

Inclusion required a diagnosis of PsO, PsA with axial involvement, and an age ≥18 years. Patients previously enrolled in other arms of GESPIC were excluded to ensure cohort integrity and avoid data overlap. A formal sample size calculation was not conducted, as this was an exploratory cohort study.

### Study visits and assessments

Baseline assessments were conducted at the initial visit, followed by evaluations every six months for the first two years and annually thereafter (Supplementary Figure 1). The study protocol included a comprehensive evaluation of PsA with axial involvement (Supplementary Table 1). Documentation encompassed details of the PsO diagnosis, including age at onset, subtype, and nail involvement. The presence and severity of current PsO were quantified using the Psoriasis Area and Severity Index (PASI).^32^ Back pain was a central focus, with data collected on its occurrence (duration of back pain exceeding three months), anatomical localization (e.g., presence of alternating buttock pain or localized axial pain), and clinical features (e.g., morning stiffness lasting longer than 30 minutes and improvement with NSAID [nonsteroidal anti-inflammatory drug] intake).^33^ In addition, ‘inflammatory back pain’ (IBP) was evaluated according to the ASAS criteria^34^ (onset before the age of 40 years, variation of symptoms with rest or movement [improvement or worsening], sudden versus gradual onset of back pain, and the presence of nocturnal pain). The assessment enabled study physicians to perform a global evaluation of whether the pain exhibited features consistent with IBP. Spinal mobility was assessed using the Bath Ankylosing Spondylitis Metrology Index (BASMI).^35^ Peripheral manifestations were systematically evaluated, including assessment of current and historical dactylitis, arthritis, and enthesitis. A 66/68 joint count was performed to calculate the Disease Activity in Psoriatic Arthritis (DAPSA) score,^36^ and enthesitis was additionally assessed using the full 0–29 enthesitis count.^35^ Furthermore, associated conditions such as anterior uveitis and chronic inflammatory bowel disease, were documented.

### Laboratory and biosample collection

A comprehensive panel of laboratory investigations was obtained, including inflammatory markers (C-reactive protein [CRP], erythrocyte sedimentation rate [ESR]) and metabolic parameters (Supplementary Table 1). HLA-B27 testing and rheumatoid factor assessment were performed when not previously available. Urine and stool samples (both native and stabilized) were collected and are stored at −80 °C. In addition, patient serum and peripheral blood mononuclear cells (PBMCs) were cryopreserved for subsequent analyses.

### Patient-reported outcomes and socioeconomic data

Patient-reported outcomes were systematically collected using validated instruments, including the Patient-Acceptable Symptom State (PASS), Bath Ankylosing Spondylitis Disease Activity Index (BASDAI), Bath Ankylosing Spondylitis Functional Index (BASFI), ASAS Health Index (ASAS-HI), Health Assessment Questionnaire–Disability Index (HAQ-DI), PainDetect, Widespread Pain Index (WPI), and the Food Frequency Questionnaire (FFQ).^35,37–39^ Additionally, socioeconomic factors were assessed, alongside detailed histories of nicotine and alcohol consumption.

### Treatment documentation

All treatments targeting both cutaneous and musculoskeletal manifestations were systematically documented, including local and systemic therapies.

### Imaging protocol and image interpretation

Conventional radiographs were obtained, including *anterior–posterior* (AP) and lateral views of the lumbar spine, lateral views of the cervical spine, and sacroiliac joint (SIJ) imaging according to Ferguson’s method. Additionally, MRI of the entire spine and SIJ was performed using a 3.0 Tesla scanner following a standardized protocol (Supplementary Text 1), including sagittal T1-weighted turbo spin echo (T1 TSE) and short tau inversion recovery (STIR) sequences of the cervical, thoracic, and lumbar spine. Oblique coronal T1 TSE and STIR sequences of the SIJs were obtained, with at least 18 contiguous slices ensuring complete coverage, particularly of the ventral pelvic region. Sagittal sequences were reconstructed to enable continuous multiplanar assessment of the entire spine.

Conventional radiographs and MRI examinations were reviewed locally in weekly multidisciplinary meetings involving the treating rheumatologists and a musculoskeletal radiologist with specific expertise in PsA and axSpA. Imaging findings were determined by consensus as part of the overall clinical and imaging evaluation. Conventional radiographs were graded according to the modified New York (mNY) grading system. MRI examinations were assessed qualitatively for the presence or absence of typical active inflammatory and structural lesions, both for the SIJs and the spine.

As no disease-specific MRI definitions for axial PsA are currently established, interpretation was informed by the lesion terminology and general principles of the updated ASAS MRI Working Group definitions,^40^ without formally applying a formal evaluation algorithm. Active inflammatory lesions primarily comprised bone marrow oedema in typical subchondral or periarticular locations in the SIJs and at vertebral corners or endplates in the spine. Structural lesions included erosions, fat lesions or metaplasia, backfill, sclerosis, bone proliferation, and ankylosis, as applicable to the SIJs or spine. Lesions were classified as compatible with inflammatory axial involvement based on their morphology, anatomical distribution, and the multidisciplinary consensus assessment. No formal quantitative MRI scoring system was applied, and imaging assessments were not yet performed as a blinded central reading.

### Description of subgroups

For the subgroup analyses, the following definitions were applied. The subgroup with isolated spinal involvement (“*isolated spine*” group) included only patients who showed typical active inflammatory and/or structural lesions on MRI of the spine, but had neither typical active inflammatory or structural lesions on MRI of the SIJs nor radiographic sacroiliitis fulfilling the mNY criteria on conventional radiographs. The “others” group comprised all remaining patients with definite SIJ involvement, defined as radiographic sacroiliitis fulfilling the mNY criteria and/or typical active inflammatory or structural lesions on SIJ MRI, irrespective of whether concomitant spinal lesions were present.

Unilateral SIJ abnormalities on conventional radiographs were defined as structural changes limited to one SIJ, corresponding to grade 2 or higher according to the mNY grading system. Thus, isolated unilateral grade 2 radiographic SIJ abnormalities could be present in isolated spine group, whereas unilateral grade 3 or 4 changes or bilateral grade 2 or higher changes, which fulfil the mNY criteria, were not permitted. Asymmetric sacroiliitis was defined as a difference of two grades or more between the left and right SIJ on conventional radiographs, based on the mNY grading system.

### Statistical analysis

The analysis was primarily descriptive and focused on characterizing the baseline clinical and imaging features of patients with imaging-confirmed axial PsA.

Categorical variables are presented as number and percentage, n (%). Continuous variables are presented as mean ± standard deviation (SD) or as median with interquartile range (Q1, Q3), as appropriate according to their distributional characteristics. For subgroup comparisons between subgroups, categorical variables were compared using Pearson’s chi-square test or Fisher’s exact test, as appropriate depending on the distribution of cell counts and expected frequencies. No imputation was performed regarding missing data.

The distribution of continuous variables included in subgroup comparisons was reassessed systematically and separately within the two groups (“isolated spine” and “others”). Normality was evaluated using the Shapiro–Wilk test, supported by consideration of the overall distributional pattern and degree of skewness. Continuous variables with clearly non-normal distributions were summarized as median (Q1, Q3). For comparisons of continuous variables between groups, the Mann–Whitney U test was used. For the variables with highly zero-inflated distribution, median (Q1, Q3) summaries were considered insufficiently informative, as they frequently resulted in values such as 0.0 (0.0, 0.0) in both groups. Therefore, these variables are presented descriptively as mean ± SD for better readability, while the corresponding subgroup comparisons were still performed using the Mann–Whitney U test.

In addition to the descriptive subgroup comparisons, we performed an exploratory logistic regression analysis to assess factors associated with isolated spine involvement. The dependent variable was isolated spine involvement (yes/no). Univariable logistic regression analyses were first performed for variables that showed significant differences in the subgroup comparisons and were considered clinically relevant, while avoiding variables that were directly linked to the imaging-based definition of isolated spine involvement. Odds ratios (ORs) with 95% confidence intervals (CIs) were calculated. Subsequently, an exploratory multivariable logistic regression model was fitted with selected variables on the basis of their relevance.

Analyses were performed using R (R Foundation for Statistical Computing, Vienna, Austria), with two-sided p < 0.05 considered statistically significant.

The reporting of this study conforms to the Strengthening the Reporting of Observational Studies in Epidemiology (STROBE) statement^41^ (Supplementary Table 3).

## Results

### Clinical characteristics and patient reported outcomes

A total of 107 patients with axial-PsA were enrolled (Tables 1–3). The age distribution was centred in middle adulthood, with a marginally higher representation of female subjects, and overweight/obesity was common, with about one-third fulfilling criteria for obesity. As current or past psoriasis (PsO) was mandatory for inclusion, all patients had a history of PsO; almost 80% had active skin disease with generally low PASI scores, and more than one-third had nail involvement. PsO and PsA were long-standing, whereas axial symptoms had a somewhat shorter duration (Table 1).

**Table 1.** Demographics, clinical characteristics, disease activity, treatment.

Variable	Overall N = 107	Isolated spine N = 20	Others N = 87	p-value (isolated spine vs. Others)
**Demographic characteristics**				
Female sex	59 (55.1)	15 (75.0)	44 (50.6)	**0.048**
Age, years	44.8 ± 13.0	52.9 ± 12.3	43.0 ± 12.6	**0.002**
Body mass index, kg/m^2^ (n=106)	27.6 ± 6.6	28.4 ± 5.2	27.4 ± 6.9	0.28
Smoking ever	73 (68.2)	16 (80.0)	57 (65.5)	0.21
**Disease-, EMMs- and family history**				
Age at psoriasis onset, years	33.6 ± 16.2	46.5 ± 12.5	30.6 ± 15.5	**<0.001**
Psoriasis symptom duration, years	10.2 (3.8, 22.5)	7.1 (3.3, 15.8)	10.4 (4.7, 23.6)	0.23
PsA symptom duration, years	5.0 (1.1, 12.9)	5.0 (1.0, 10.7)	5.1 (1.3, 14.3)	0.47
Duration since diagnosis of axial-PsA, years	5.6 (1.5, 18.3)	4.9 (1.0, 8.0)	6.3 (1.7, 22.5)	0.13
History of Uveitis	15 (14.0)	0 (0.0)	15 (17.2)	**0.068**
History of IBD	7 (6.5)	3 (15.0)	4 (4.6)	0.12
Family history of psoriasis	37 (34.6)	10 (50.0)	27 (31.0)	0.11
Family history of PsA	7 (6.5)	1 (5.0)	6 (6.9)	>0.99
Family history of axial SpA	11 (10.3)	0 (0.0)	11 (12.6)	0.12
**Physical Examination and disease activity**				
Current psoriasis	84 (78.5)	13 (65.0)	71 (81.6)	0.13
Nail involvement, ever	46 (43.0)	11 (55.0)	35 (40.2)	0.23
PASI score at the current visit	1.5 (0.4, 3.9)	0.9 (0.0, 3.3)	1.5 (0.4, 4.7)	0.26
Any peripheral manifestation, ever	62 (57.9)	8 (40.0)	54 (62.1)	**0.071**
Any peripheral manifestation, current	49 (45.8)	10 (50.0)	39 (44.8)	0.68
Peripheral arthritis, ever	49 (45.8)	5 (25.0)	44 (50.6)	**0.038**
Peripheral arthritis, current	24 (22.4)	1 (5.0)	23 (26.4)	**0.040**
TJC at the current visit*	0.5 ± 1.2	0.1 ± 0.4	0.6 ± 1.3	**0.043**
SJC at the current visit*	0.5 ± 1.2	0.1 ± 0.4	0.6 ± 1.3	**0.043**
Enthesitis, ever	36 (33.6)	6 (30.0)	30 (34.5)	0.70
Enthesitis, current	33 (30.8)	10 (50.0)	23 (26.4)	**0.040**
Dactylitis, ever	16 (15.0)	1 (5.0)	15 (17.2)	0.30
Dactylitis, current	11 (10.3)	0 (0.0)	11 (12.6)	0.12
PGA, 0-10 NRS	5.1 ± 2.7	5.4 ± 3.0	5.1 ± 2.6	0.62
PhGA, 0-10 NRS	4.2 ± 2.0	3.9 ± 2.0	4.2 ± 2.0	0.42
DAPSA	11.8 ± 6.1	10.6 ± 6.8	12.0 ± 5.9	0.39
BASDAI – Question 2	5.7 ± 2.6	5.8 ± 2.7	5.7 ± 2.6	0.84
BASDAI	4.7 ± 2.0	4.6 ± 2.2	4.7 ± 2.0	0.77
ASDAS	2.7 ± 1.0	2.6 ± 1.0	2.8 ± 1.0	0.41
BASFI	3.6 ± 2.5	3.8 ± 2.5	3.6 ± 2.5	0.77
BASMI (n=105)	2.8 (2.0, 3.6)	2.8 (2.6, 3.6)	2.6 (1.8, 3.4)	0.23
ASAS Health-Index	7.0 ± 3.9	7.9 ± 3.8	6.9 ± 4.0	0.30
**Laboratory**				
CRP, mg/L	2.3 (1.0, 9.0)	2.1 (1.2, 5.0)	2.3 (1.0, 11.8)	0.72
Elevated CRP (≥5 mg/L)	35 (32.7)	5 (25.0)	30 (34.5)	0.42
HLA-B27 Positivity	52 (48.6)	4 (20.0)	48 (55.2)	**0.005**
Rheumatoid factor Positivity (n=103)	9 (8.7)	3 (15.0)	6 (7.2)	0.37
**Treatment**				
Physiotherapy, ever	61 (57.0)	10 (50.0)	51 (58.6)	0.48
NSAID for PsA	83 (77.6)	14 (70.0)	69 (79.3)	0.38
csDMARDs, ever	46 (43.0)	10 (50.0)	36 (41.4)	0.48
b- or ts-DMARDs, ever	37 (34.6)	4 (20.0)	33 (37.9)	0.13
b- or ts-DMARDs, current	15 (14.0)	1 (5.0)	14 (16.1)	0.29

Variables are presented as mean ± SD, median (Q1, Q3), or number (%) as appropriate.

P values were calculated using Pearson’s chi-square test or Fisher’s exact test for categorical variables, and Student’s t-test or Mann–Whitney U test for continuous variables, as appropriate.

ASAS: Assessment of SpondyloArthritis International Society, ASDAS: Axial Spondyloarthritis Disease Activity Score, BASDAI: Bath Ankylosing Spondylitis Disease Activity Index, BASFI: Bath Ankylosing Spondylitis Functional Index, BASMI: Bath Ankylosing Spondylitis Metrology Index, BP: Back pain, b- or ts-DMARDs: biological or targeted synthetic Disease-Modifying Antirheumatic Drugs, CRP: C-reactive protein, csDMARDs: Conventional synthetic Disease-Modifying Antirheumatic Drugs, DAPSA: Disease Activity in Psoriatic Arthritis, EMMs: Extra-musculoskeletal manifestations, ESR: Erythrocyte Sedimentation Rate, HLA-B27: Human Leukocyte Antigen B27, IBP: Inflammatory back pain, IBD: Inflammatory bowel disease, NRS: Numerical Rating Scale, NSAID: Nonsteroidal antiinflammatory drugs, PASI: Psoriasis Area and Severity Index, PGA: Patient Global Assessment, PhGA: Physician Global Assessment of Musculoskeletal and Skin, PsA: Psoriatic arthritis, SIJ: Sacroiliac Joint, SJC: Swollen Joint Count, SpA: Spondyloarthritis, TJC: Tender Joint Count.The bold values are used to highlight significant p-value results.

*These variables showed highly zero-inflated distributions; median (Q1, Q3) summaries were therefore uninformative, and they are presented as mean ± SD, while p values were derived from the Mann–Whitney U test.

**Table 2.** Back pain characteristics.

Variable	Overall N = 107	Isolated spine N = 20	Others N = 87	p-value (isolated spine vs. Others
Current BP	103 (96.3)	20 (100.0)	83 (95.4)	>0.99
**Location of current BP**				
Buttocks/sacroiliac joints (n=103)	73 (70.9)	8 (40.0)	65 (78.3)	**<0.001**
Total Spine (n=103)	101 (98.1)	20 (100.0)	81 (97.6)	>0.99
Cervical spine (n=103)	44 (42.7)	11 (55.0)	33 (39.8)	0.22
Thoracic spine (n=103)	58 (56.3)	15 (75.0)	43 (51.8)	**0.060**
Lumbar spine (n=103)	86 (83.5)	16 (80.0)	70 (84.3)	0.74
**IBP characteristics**				
Duration of current BP, years (n=103)	9.7 (2.7, 20.2)	9.5 (5.0, 14.8)	9.8 (2.3, 20.4)	0.92
Age of current BP onset <45 years (n=103)	90 (87.4)	12 (60.0)	78 (94.0)	**<0.001**
Current BP, Insidious onset (n=103)	86 (83.5)	18 (90.0)	68 (81.9)	0.51
Morning stiffness > 30min (n=103)	72 (69.9)	12 (60.0)	60 (72.3)	0.28
Improvement through exercise (n=103)	70 (68.0)	9 (45.0)	61 (73.5)	**0.014**
No improvement with rest (n=103)	76 (73.8)	12 (60.0)	64 (77.1)	0.12
Night pain (n=103)	80 (77.7)	16 (80.0)	64 (77.1)	>0.99
Current BP is better with NSAIDs (n=97)	82 (84.5)	12 (66.7)	70 (88.6)	**0.031**
Current BP, IBP according to global evaluation (n=103)	81 (75.7)	10 (50.0)	71 (81.6)	**0.007**
Current BP, IBP according to ASAS criteria (n=103)	65 (63.1)	8 (40.0)	57 (68.7)	**0.017**

Variables are presented as median (Q1, Q3) or number (%) as appropriate. P values were calculated using Pearson’s chi-square test or Fisher’s exact test for categorical variables, and Student’s t-test or Mann–Whitney U test for continuous variables, as appropriate. ASAS: Assessment of SpondyloArthritis International Society, BP: Backpain, IBP: inflammatory backpain, NSAID: Nonsteroidal antiinflammatory drug.The bold values are used to highlight significant p-value results.

**Table 3.** Assessment of imaging (SIJ and Spine X-Ray and MRI) and classification criteria.

Variable	Overall N = 107	Isolated spine N = 20	Others N = 87	p-value (isolated spine vs. Others)
mNYc Positive conventional radiographs (n=105)	48 (45.7)	0 (0.0)	48 (55.8)	**<0.001**
Unilateral SIJ abnormalities on conventional radiographs (n=105)	65 (61.9)	4 (21.1)	61 (70.9)	**<0.001**
Asymmetrical sacroiliitis on conventional radiographs (n=105)	14 (13.3)	2 (10.5)	12 (14.0)	>0.99
**Any Active inflammatory lesions in SIJ-MRI** (n=105*)	56 (53.3)	0 (0.0)	56 (65.9)	**<0.001**
**Active inflammatory lesions in SIJ-MRI** (n=105*)	​	​	​	**<0.001**
No	49 (46.7)	20 (100.0)	29 (34.1)	​
Unilateral	21 (20.0)	0 (0.0)	21 (24.7)	​
Bilateral	35 (33.3)	0 (0.0)	35 (41.2)	​
**Any Structural lesions in SIJ-MRI** (n=105*)	77 (73.3)	0 (0.0)	77 (90.6)	**<0.001**
**Structural lesions in SIJ-MRI** (n=105*)	​	​	​	**<0.001**
No	28 (26.7)	20 (100.0)	8 (9.4)	​
Unilateral	8 (7.6)	0 (0.0)	8 (9.4)	​
Bilateral	69 (65.7)	0 (0.0)	69 (81.2)	​
**Any Active lesions in Spine-MRI** (n=105*)	63 (60.0)	19 (95.0)	44 (51.8)	**<0.001**
**Any Structural lesions in Spine-MRI** (n=105*)	56 (53.3)	18 (90.0)	38 (44.7)	**<0.001**
**Fullfillment of ASAS or CASPAR Criteria** * *	​	​	​	**<0.001**
ASAS & CASPAR Negative	5 (4.7)	4 (20.0)	1 (1.1)	​
Only ASAS Positive	13 (12.1)	1 (5.0)	12 (13.8)	​
Only CASPAR Positive	24 (22.4)	12 (60.0)	12 (13.8)	​
ASAS & CASPAR Positive	65 (60.7)	3 (15.0)	62 (71.3)	​

The variables are presented as mean ± SD, or as number (%) unless otherwise indicated.

ASAS: Assessment of SpondyloArthritis International Society, CASPAR: Classification Criteria for Psoriatic Arthritis, mNYc: Modified New York Criteria, MRI: Magnetic Resonance Imaging, SIJ: Sacroiliac Joint, Asymmetrical Sacroiliitis: Asymmetrical sacroiliitis, according to the modified New York grading system, is defined as a difference of two or more grades between the left and right sacroiliac joints on conventional radiographs, Unilateral SIJ abnormalities by conventional radiographs: structural changes limited to one SIJ, corresponding to grade 2 or higher according to the mNY grading system.The bold values are used to highlight significant p-value results.

*The total number of MRI examinations was limited to n = 105, as one patient was unable to undergo MRI due to claustrophobia and another due to the presence of a pain catheter.

**The p value refers to a global χ^2^ test across all four mutually exclusive ASAS/CASPAR categories. Inspection of cell frequencies indicates that the statistical difference is mainly driven by the markedly higher prevalence of dual ASAS & CASPAR positivity in others, and CASPAR-only positivity in only spine group. No formal pairwise post-hoc tests were performed.

Most patients were using NSAIDs at baseline, and approximately 40% had previously received csDMARDs. About one-third had ever been treated with bDMARDs or tsDMARDs, with only a minority on ongoing advanced therapy at the time of inclusion. Extra-musculoskeletal manifestations (uveitis, inflammatory bowel disease) and a positive family history of PsO, PsA or axSpA were present in a relevant minority of patients (Table 1).

Current back pain was reported by almost all patients and most frequently involved the lumbar spine, followed by thoracic and cervical segments; buttock and sacroiliac joint (SIJ) pain were also common. Classical IBP features were frequent (onset before 45 years, insidious onset, morning stiffness, improvement with exercise and NSAIDs, and no relief with rest). Based on global physician evaluation, 78.6% were judged to have IBP, whereas 63.1% fulfilled the ASAS IBP criteria (Table 2).

More than half of the cohort reported any history of peripheral manifestations (arthritis, enthesitis or dactylitis), and nearly half had active peripheral involvement at baseline. Composite indices indicated, on average, moderate disease activity and impairments in function and health status (DAPSA, BASDAI, ASDAS, BASFI, BASMI, ASAS-HI; Table 1). Elevated CRP (≥5 mg/L) was present in about one-third of patients, and approximately half were HLA-B27 positive, whereas rheumatoid factor positivity was infrequent, occurring in fewer than 10% of patients.

### Imaging evaluation

According to the radiographic evaluation, 45.7% of patients fulfilled the mNY criteria for radiographic sacroiliitis. Unilateral SIJ abnormalities on conventional radiographs were present in 61.9%, and asymmetric sacroiliitis was observed in 13.3% (Table 3). Among patients in the isolated spine group with available radiographs, four had unilateral grade 2 SIJ abnormalities; however, none fulfilled the mNY criteria or had active inflammatory or structural SIJ lesions on MRI.

**Figure 1.**
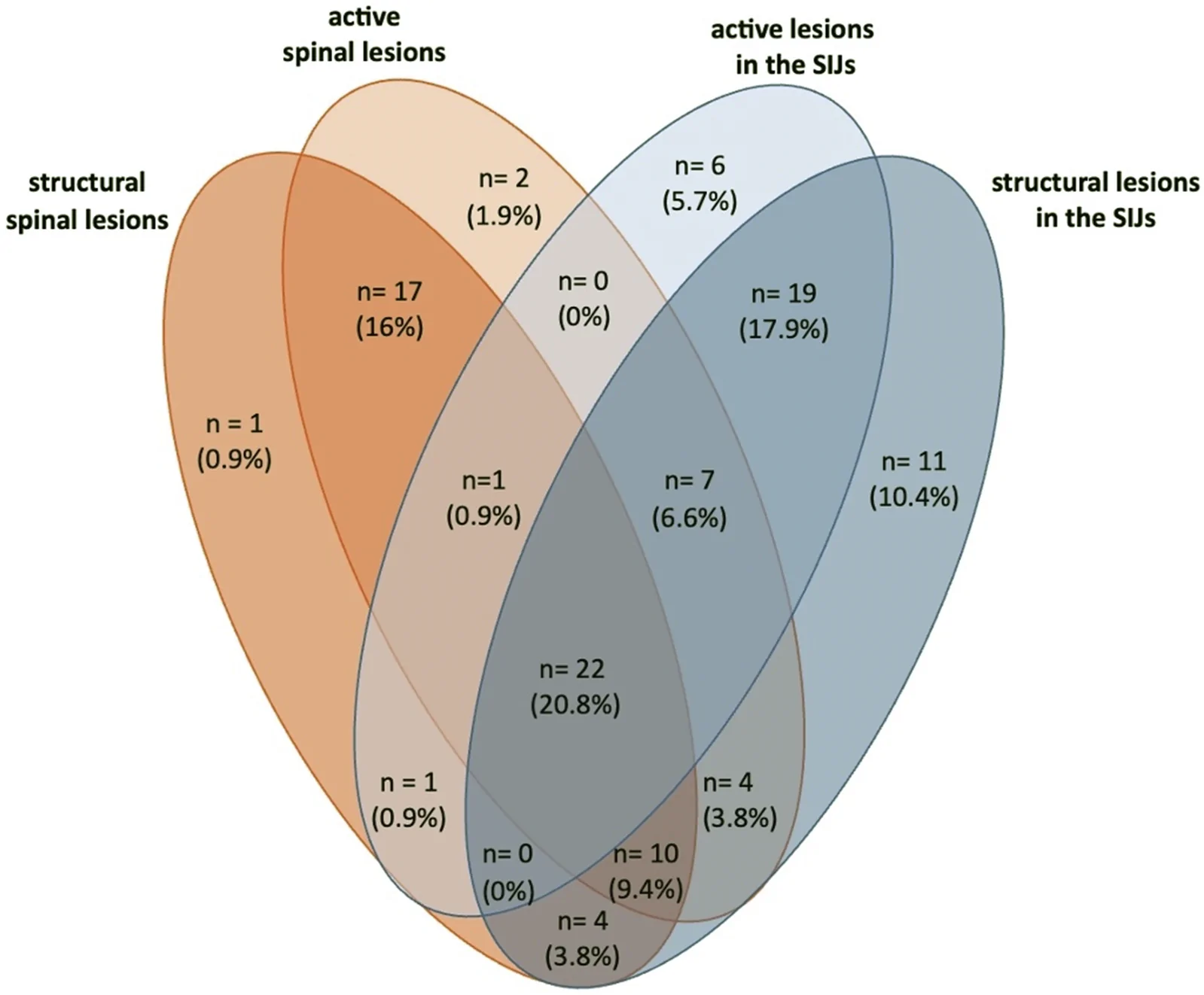
Venn-diagram MRI imaging. MRI: Magnetic Resonance Imaging, SIJ: Sacroiliac Joint, n=105*. * The total number of MRI examinations was limited to n = 105, as one patient was unable to undergo MRI due to claustrophobia and another due to the presence of a pain catheter.

On MRI, as shown in Figure 1 and Table 3, active inflammatory lesions in the SIJs were present in 53.3% (20.0% unilateral, 33.3% bilateral), while structural SIJ lesions were observed in 73.3% (7.6% unilateral, 65.7% bilateral). In the spine, active inflammatory and structural lesions were found in 60.0% and 53.3% of patients, respectively.

### Application of classification criteria for axSpA and/or PsA

**Figure 2.**
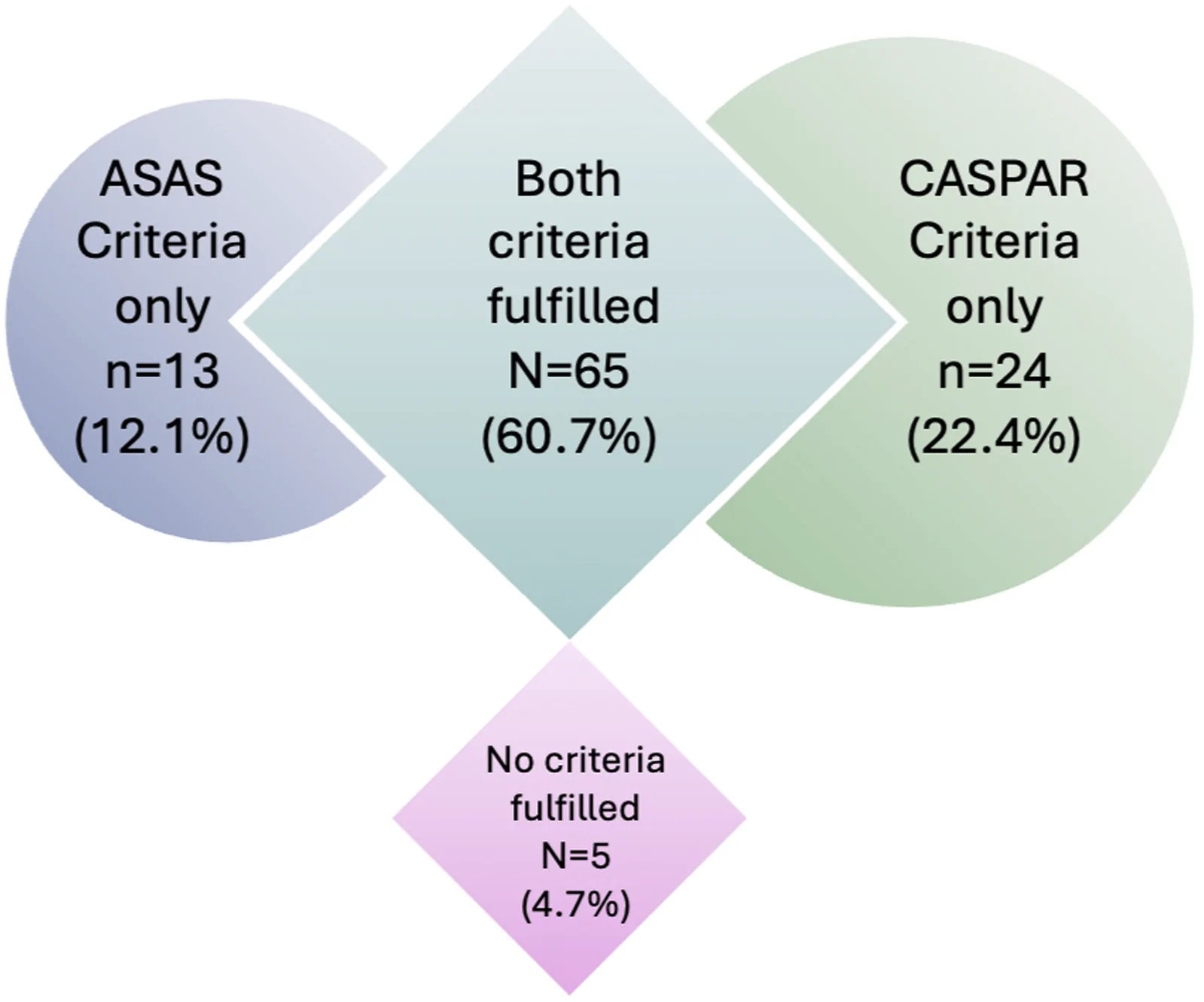
Fulfillment of ASAS- and CASPAR-criteria. ASAS: Assessment of SpondyloArthritis international Society, CASPAR: Classification Criteria for Psoriatic Arthritis.

Concerning existing classification criteria for axial SpA (ASAS criteria) and for PsA (CASPAR criteria) most patients (60.7%) simultaneously fulfilled both ASAS and CASPAR criteria. An additional 22.4% met only CASPAR, and 12.1% only met ASAS criteria, as shown in Figure 2 and Table 3. A minority of 5 patients (4.7%) did not fulfill either set of classification criteria, as shown in Supplementary Tables 2 and 3. In regards of the ASAS classification criteria for axSpA, four out of five patients did not meet the entry criterion of experiencing back pain before the age of 45. The one patient who did meet this criterion was HLA-B27 negative and did not show compatible imaging findings of the SIJ.

### Comparison of sub-groups

Comparative analyses of baseline characteristics between patients with isolated spinal involvement and all other patients included all 107 individuals: 20 (18.7%) constituted the “*isolated spine*” group (axial involvement limited to the spine), and 87 (81.3%) the “*others*” group (sacroiliac involvement with or without spinal disease). Key demographic and clinical features are summarized in Table 1. Patients in the “*isolated spine*” group were more often female, were older at inclusion, and had a later onset of PsO compared with the “*others*” group. Patterns of peripheral disease also differed: peripheral arthritis was less frequent, while enthesitis was more common in the “*isolated spine*” group, and dactylitis occurred only in the “*others*” group. Measures of disease activity, function and health status (BASDAI, ASDAS, BASFI, BASMI, ASAS-HI) were broadly similar between groups. CRP levels tended to be lower and HLA-B27 positivity was markedly less common in the “*isolated spine*” group (20.0% vs. 55.2%), although most other treatment-related variables did not differ substantially (Table 1).

Back-pain characteristics are detailed in Table 2. All patients in the “*isolated spine*” group reported current back pain, similar to the “*others*” group. However, back pain onset before 45 years and improvement with exercise or NSAIDs were less frequently reported in the “*isolated spine*” group. Consequently, IBP—as judged by global physician assessment (50.0% vs. 85.5%) or by fulfilment of the ASAS IBP criteria (40.0% vs. 68.7%)—was significantly less common in the “*isolated spine*” group, while other IBP features were largely comparable between groups.

Marked differences were observed in imaging findings (Table 3). None of the patients in the “*isolated spine*” group fulfilled the mNY criteria for sacroiliitis or showed active inflammatory or structural lesions in the SIJ on MRI, whereas these features were present in the majority of patients in the “*others*” group. In contrast, active and structural spinal lesions on MRI were detected in almost all patients with isolated spinal involvement but in only about half of the “*others*” group.

### Uni- and multivariable analyses of factors associated with isolated spine involvement

In exploratory univariable logistic regression analyses, isolated spine involvement was associated with older age, lower HLA-B27 positivity, less frequent buttock/sacroiliac joint pain, less frequent onset of current back pain before age 45 years, and less frequent inflammatory back pain according to both global evaluation and ASAS criteria, whereas current enthesitis showed a positive direction of association. Female sex also showed a positive direction of association, while current peripheral arthritis showed an inverse direction of association (Table 4).

**Table 4.** Univariable and multivariable logistic regression for factors associated with isolated spine involvement.

Variable	Univariable logistic regression		Multivariable logistic regression	
	UV OR (95% CI)	UV p-value	MV OR (95% CI)	MV p-value
Age, years	1.06 (1.02, 1.11)	0.004	-	-
Female sex	2.93 (0.98, 8.77)	0.054	1.70 (0.43, 6.78)	0.453
HLA-B27 positivity	0.20 (0.06, 0.66)	0.008	0.41 (0.10, 1.63)	0.208
Current BP, buttocks/sacroiliac joints	0.18 (0.07, 0.52)	0.001	-	-
Age of current BP onset <45 years	0.10 (0.03, 0.34)	<0.001	0.16 (0.03, 0.82)	0.028
Current BP, IBP according to global evaluation	0.23 (0.08, 0.63)	0.005	0.32 (0.08, 1.25)	0.101
Current BP, IBP according to ASAS criteria	0.30 (0.11, 0.83)	0.021	-	-
Peripheral arthritis, current	0.15 (0.02, 1.16)	0.068	0.14 (0.02, 1.34)	0.088
Enthesitis, current	2.78 (1.03, 7.55)	0.044	4.46 (1.23, 16.14)	0.023

ASAS: Assessment of SpondyloArthritis International Society; BP: back pain; CI: confidence interval; HLA-B27: human leukocyte antigen B27; IBP: inflammatory back pain; OR: odds ratio; MV: Multivarible; UV: Univariable.

In the exploratory multivariable model, younger age at onset of current back pain remained inversely associated with isolated spine involvement (OR 0.16, 95% CI 0.03–0.82), whereas current enthesitis remained positively associated (OR 4.46, 95% CI 1.23–16.14). The corresponding estimates for female sex (OR 1.70, 95% CI 0.43–6.78), HLA-B27 positivity (OR 0.41, 95% CI 0.10–1.63), current inflammatory back pain according to global evaluation (OR 0.32, 95% CI 0.08–1.25), and current peripheral arthritis (OR 0.14, 95% CI 0.02–1.34) were attenuated in the multivariable model (Table 4).

## Discussion

The primary objective of this study was to observe and characterize a well-defined, prospective cohort of patients with axial-PsA, with a particular focus on imaging morphology. It is important to note that this report is limited to the baseline analysis of an ongoing longitudinal study, however it still provides valuable insights. One of the key findings from the imaging data is that approximately 20% of patients demonstrated isolated spinal involvement without concomitant SIJ changes in MRI or x-ray. This observation is clinically significant, as it suggests a morphological pattern that may help distinguish axial-PsA from axSpA, where axial involvement typically originates in the SIJ and is therefore predominantly localized to this region.^42,43^ The higher proportion of female patients and lower HLA-B27 positivity in our cohort compared with axSpA highlights phenotypic differences and supports the notion that axial-PsA constitutes a distinct clinical entity. In addition, approximately 60% of patients fulfilled both the CASPAR and ASAS classification criteria, indicating that currently available frameworks for PsA and axSpA are insufficient to specifically identify this distinct subgroup of patients with axial-PsA.

Axial involvement in PsA has historically been investigated predominantly through retrospective studies, often as secondary analyses of broader PsA or SpA cohorts. Such studies have provided valuable insights into prevalence, imaging and clinical characteristics but are inherently limited by heterogeneous inclusion criteria, non-standardized imaging protocols, and the absence of longitudinal follow-up data.^12,44,45^ Findings from the recent Greek multicenter PsA cohort by Vassilakis et al. (2025) are broadly consistent with our results, reporting axial involvement in approximately 25% of patients and isolated spinal involvement in about 30% of these cases.^12^ Similar to our data, peripheral arthritis occurred less frequently among patients with axial involvement. However, combined sacroiliac and spinal involvement was more common in our cohort (45.8%) compared to 26% in the Greek study population. Patients in our cohort were also younger (mean 45 years vs. 55 years), while sex distribution, BMI, and family history of PsA were similar. HLA-B27 positivity was markedly higher in our cohort (50% vs. 13%), suggesting possible genetic or methodological differences. Importantly, our study complements the Greek cohort by providing a prospective design and systematic imaging with both MRI and radiographs in all patients, allowing for a more detailed characterization of axial involvement.

Similarly, data from the REGISPONSER cohort analyzed by Michelena et al. (2022) demonstrated axial involvement in approximately 30% of PsA patients, consistent with both our findings and those of the Greek cohort.^44^ Unlike our prospective study, REGISPONSER was retrospective, but demographic features such as age and BMI were broadly similar. HLA-B27 positivity was lower in that cohort (30%), although data on HLA-B27 status were available for only about 80% of patients. In contrast, the prevalence of IBP and patient-reported outcomes, including BASDAI and BASFI, closely mirrored our findings, indicating a comparable disease burden despite differences in study design.

In line with these observations, Kwok et al. (2022) reported an association between HLA-B27 positivity and isolated axial-PsA without peripheral manifestations in the Toronto PsA cohort.^45^ These findings align with our observation of elevated HLA-B27 positivity in patients with axial involvement of PsA and relatively low rates of enthesitis, dactylitis, and peripheral arthritis. Future longitudinal follow-up will be important to determine whether HLA-B27 predicts progression from isolated axial involvement to combined axial and peripheral disease, as suggested by previous studies.

An exploratory logistic regression analysis was additionally performed to examine factors associated with isolated spine involvement. In the univariable analyses, isolated spine involvement showed directional associations with older age, female sex, lower HLA-B27 positivity, less typical inflammatory back pain features, lower frequency of peripheral arthritis, and a higher frequency of enthesitis. In the multivariable model, younger age at onset of current back pain and current enthesitis remained the variables most consistently associated with this phenotype, whereas the associations with female sex, HLA-B27, inflammatory back pain according to global evaluation, and peripheral arthritis were attenuated. From a clinical perspective, these findings may suggest that patients with isolated spinal involvement represent a subgroup with a less typical axial SpA-like clinical presentation and, potentially, a different balance between axial and peripheral disease domains. However, these analyses must be interpreted with considerable caution. The number of patients with isolated spine involvement was small, the model was exploratory, and the present data are cross-sectional. Therefore, these results should not be interpreted as evidence of causal relationships, but rather as hypothesis-generating observations that may inform future studies in independent and longitudinal cohorts.

When analyzing the subgroup of five patients who did not fulfil either the ASAS or CASPAR classification criteria, the results were consistent with those reported by Paci et al.^46^ Most of these patients did not meet the ASAS entry criterion of back pain onset before the age of 45 years. Furthermore, none of them showed radiographic sacroiliitis, as four out of five belonged to the “*isolated spine*”-group. This supports the notion that late-onset axial disease in psoriatic arthritis may be under-recognized when the ASAS criteria are applied.

A further point requiring careful interpretation is the occurrence of low-grade unilateral radiographic SIJ abnormalities in a small number of patients classified as having isolated spinal involvement. Isolated unilateral grade 2 radiographic abnormalities, or bilateral grade 1 changes, do not fulfil the mNY criteria for radiographic sacroiliitis and were therefore not considered sufficient to define definite SIJ involvement when MRI showed neither active inflammatory nor structural SIJ lesions. This distinction is relevant because conventional radiographs of the SIJ are prone to interpretative uncertainty, particularly in the setting of low-grade or unilateral changes, and may be affected by projectional, degenerative, or other non-inflammatory abnormalities. Accordingly, the few mNY-negative unilateral radiographic SIJ changes observed in the isolated spine group were interpreted as potentially false-positive nonspecific radiographic findings rather than definite sacroiliac involvement. Nevertheless, this finding highlights the challenges of defining imaging phenotypes in axial PsA and underscores the need for standardized, disease-specific classification criteria. Longitudinal follow-up will be important to determine whether such low-grade radiographic findings represent early or residual SIJ disease, nonspecific radiographic changes, or remain clinically irrelevant over time.

### Limitations

However, several limitations of our study should be acknowledged. First, the study was conducted at a specialized tertiary referral center for axSpA, which may have introduced selection bias and potentially limits the generalizability of our findings to broader clinical settings. This referral pattern may also explain the relatively high proportion of patients with isolated axial-PsA without peripheral manifestations, a distribution that may not reflect the typical spectrum seen in routine rheumatological practice. Moreover, peripheral joint and entheseal imaging was not systematically performed, restricting the ability to assess the full extent of peripheral musculoskeletal involvement. In addition, the absence of centralized reading for spinal imaging may have introduced inter-reader variability, potentially affecting the consistency and comparability of imaging assessments. Finally, the included PsA patients had a relatively long mean symptom duration of nearly nine years, which may have influenced the observed findings; therefore, it would be of interest to replicate these results in an independent cohort with shorter symptom duration to assess their robustness across different disease stages and to evaluate longitudinal outcomes in this specific population. The absence of a predefined sample size calculation may limit the statistical power of the analyses and should be acknowledged also as a potential limitation of this study.

Despite these limitations, the study also possesses notable strengths. Most prominently, it represents a dedicated cohort study specifically investigating the axial manifestation of PsA, thereby addressing an important unmet need in the field. A major asset is the comprehensive and systematic characterization of patients, encompassing both detailed clinical phenotyping and an extensive multimodal imaging strategy. The latter combined MRI of the SIJ and spine—performed according to a predefined standardized protocol—with conventional radiographs of the entire spine and SIJ, thereby enhancing sensitivity and specificity for detecting structural and inflammatory lesions and allowing a nuanced characterization of axial-PsA phenotypes. All patients were evaluated at a single expert center, ensuring a homogeneous assessment environment with standardized clinical and imaging protocols. Evaluations were performed by rheumatologists and radiologists with specific expertise in spondyloarthritis. The prospective design with ongoing follow-up further strengthens the validity of the observations and facilitates longitudinal assessment of disease trajectories. Moreover, conducting the study within an ASAS/GRAPPA expert center facilitated adherence to internationally recognized classification and imaging standards, thereby increasing the methodological rigor and supporting comparability of the findings. Ongoing follow-up is expected to provide further insights into disease progression and may help clarify the role of imaging in characterizing axial-PsA. Finally, this unique imaging dataset can be retrospectively leveraged to explore emerging concepts, such as the ‘*ligamentitis’* hypothesis.^18^

This study raises several important questions and highlights key directions for future research in axial-PsA. One of the most pressing gaps is the absence of validated classification criteria specific to axial-PsA, as well as a lack of consensus on characteristic imaging features that define this entity. In axial-PsA, IBP is less prevalent than in axSpA, and neither rheumatologist-assessed IBP nor established IBP criteria reliably capture axial involvement, underscoring the need for disease-specific outcome measures and activity assessment tools tailored to axial-PsA that differ from those used in axSpA.^16,47^

An important future direction relates to the ongoing efforts of the AXIS study group, which is currently developing classification criteria for axial-PsA.^25^ At present, no validated definition or classification framework exists for this disease domain, highlighting the relevance of cohorts with comprehensive imaging protocols. To our knowledge, this cohort is the only study to date with imaging data comparable in scope to AXIS,^25^ including complete spinal and SIJ MRI in addition to conventional radiography of both SIJ and spine. This unique dataset will be well positioned to inform and potentially support the validation of future classification criteria once established. Moreover, follow-up assessments and longitudinal imaging will enable re-evaluation of disease characteristics as classification criteria evolve, thereby supporting future harmonization between research and clinical practice in axial-PsA.

## Conclusions

We identified isolated spinal involvement in approximately 20% of axial-PsA cases, consistent with previous reports,^12,24,48^ and uniquely supported by a complete whole-spine MRI dataset. This standardized imaging protocol enables robust morphological characterization and facilitates longitudinal monitoring.

The observed differences in the isolated spinal axial-PsA subgroup will help refine our understanding of distinct phenotypes within axial-PsA. Differences in HLA-B27 prevalence, IBP frequency, and imaging patterns compared with axSpA further highlight the need for disease-specific classification criteria.

Beyond imaging, the unique baseline dataset and associated biosamples (blood, stool, urine) provide a valuable resource for future studies on disease mechanisms. The prospective design with ongoing follow-up will enable comprehensive tracking of structural progression, treatment responses, and the natural disease course.

## Supplemental material

Supplemental material - Clinical and imaging characteristics of patients with axial psoriatic arthritis: Baseline findings from the GESPIC-axial-PsA cohort Supplemental material for Clinical and imaging characteristics of patients with axial psoriatic arthritis: Baseline findings from the GESPIC-axial-PsA cohort by Henriette Baum, Murat Torgutalp, Valeria Rios Rodriguez, Judith Rademacher, Mikhail Protopopov, Hildrun Haibel, Burkhard Muche, Kay-Geert Hermann, Denis Poddubnyy, and Fabian Proft in Therapeutic Advances in Musculoskeletal Disease

## Data Availability

Data are available upon reasonable request. Deidentified participant data can be made available after approval of a written request for scientific purposes by the corresponding author.*
