# Supplementary material for: Clinical and imaging characteristics of patients with axial psoriatic arthritis: Baseline findings from the GESPIC-axial-PsA cohort
Source: Ther Adv Musculoskelet Dis. 2026 Sep 3;18:1759720X261472985. doi: 10.1177/1759720X261472985 (PMC13546424; doi:10.1177/1759720X261472985)
Supplement: Supplementary file 1 [file sj-pdf-1-tab-10.1177_1759720X261472985.pdf]

Supplementary Figure 1

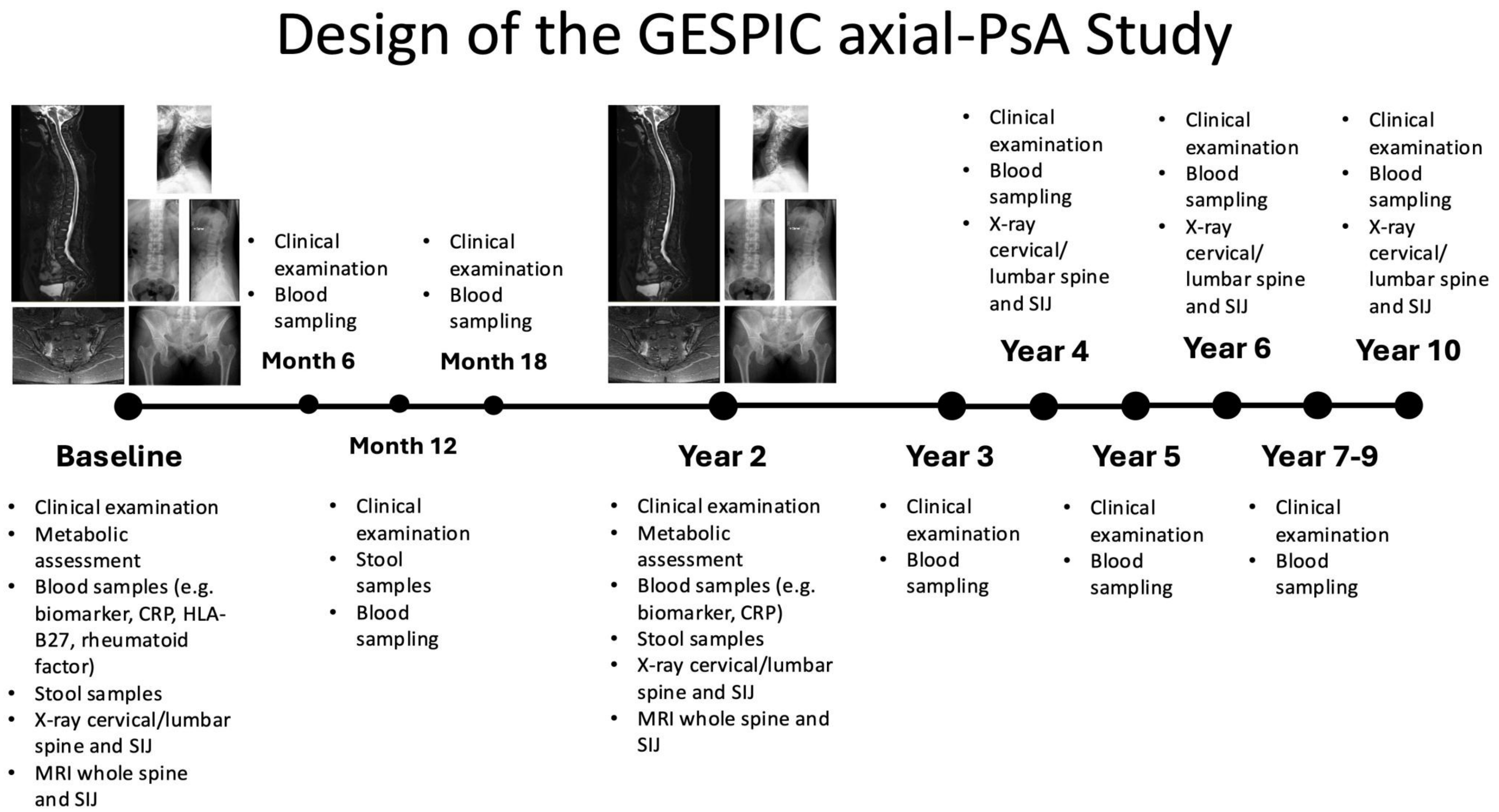

Supplementary Table 1. Study plan (Presentation according to study protocol 10 version 5.1 (Amendment 6), 11.01.2019)

Study procedure	Baseline	Month 6	Year 1	Month 18	Year 2	Year 3	Year 4	Year 5	Year 6	Year 7,8,9	Year 10
	Visit 0	Visit 1	Visit 2	Visit 3	Visit 4	Visit 6	Visit 8	Visit 10	Visit 12	Visit 14,16,18	Visit 20
Declaration of consent	X										
Inclusion and exclusion criteria	X										
Diagnosis (Re)Evaluation	X	X	X	X	X	X	X	X	X	X	X
Back pain evaluation	X	X	X	X	X	X	X	X	X	X	X
Extraarticular manifestations [a]	X	X	X	X	X	X	X	X			
Surgical history (SpA/Crohn's disease)	X	X	X	X	X	X	X	X	X	X	X
Therapy acquisition	X	X	X	X	X	X	X	X	X	X	X
Arthritis [b]	X	X	X	X	X	X	X	X	X	X	X
Enthesitis [c]	X	X	X	X	X	X	X	X	X	X	X
BASMI	X	X	X	X	X	X	X	X	X	X	X
Global physician assessment (NRS)	X	X	X	X	X	X	X	X	X	X	X
ESR, CRP	X	X	X	X	X	X	X	X	X	X	X
Blood sample	X	X	X	X	X	X	X	X	X	X	X
Stool sample	X		X		X		X		X		X
Urine sample	X	X	X	X	X	X	X	X	X	X	X
X-ray examination of the SIJ and spine [d]	X				X		X		X		X
MRI of the SIG and spine [e]	X				X						
Questionnaires: BASDAI, BASFI, Patient Global, PASS, pain, disability, socio-economic factors	X	X	X	X	X	X	X	X	X	X	X
Smoking/Alcohol Evaluation[f]	X	X	X	X	X	X	X	X	X	X	X
Metabolic evaluation	X	X	X	X	X	X	X	X	X	X	X
Psoriasis Evaluation	X	X	X	X	X	X	X	X	X	X	X

a. Uveitis, psoriasis, IBD and extraintestinal manifestations typical of IBD. b. 64-joint count. c. MASES / SPARCC scores. d. According to the clinical standard for patients with axial SpA or suspected SpA. No study-specific examination, only evaluation of routinely collected X-ray data (sacroiliac joints - pelvic overview, cervical spine - lateral only, lumbar spine - lateral and AP).e.T1-weighted sequence and STIR sequence. f. including insulin (mU/L), glucose (mg/dL), total cholesterol (mg/dL), HDL cholesterol (mg/dL), LDL cholesterol (mg/dL), triglycerides (mg/dL), and HbA1c (mmol/mol).

Supplementary Table 2. Fulfillment of the main criteria of the ASAS & CASPAR negative patients

ASAS criteria	BP ≥ 3 months	Age at onset of BP <45 years	HLA-B27	Sacroiliitis on imaging (MRI or X-ray)
**Patient 1**	?	?	X	X
**Patient 2**	?	X	X	X
**Patient 3**	?	X	X	X
**Patient 4**	?	X	X	X
**Patient 5**	?	X	X	X

CASPAR criteria	PsO (active or history)	Nail involvement	Rheumatoid factor	Daktylitis	Hand or foot plain radiography
**Patient 1**	history	negative	negative	negative	n.d
**Patient 2**	history	negative	negative	negative	n.d
**Patient 3**	history	negative	negative	negative	n.d
**Patient 4**	history	positive	positive	negative	n.d
**Patient 5**	history	negative	negative	negative	n.d

ASAS: Assessment of SpondyloArthritis international Society, CASPAR: Classification Criteria for Psoriatic Arthritis, BP: Backpain; PsO: Psoriasis; n.d.: not done; HLA-B27: Human Leukocyte Antigen B27

Supplementary Text 1. Imaging Protocol

CHARITÉ
UNIVERSITÄTSMEDIZIN BERLIN

GESPIC
GErman Spondyloarthritis Inception Cohort

Establishment of an inception cohort for spondyloarthropathies (GErman SPondyloarthritis Inception Cohort – GESPIC)

Technical protocol – MRI

MRI device: 3.0T (1.5T also possible)

Region examined: Entire spine and SIJ

Sequences:
- Localizer
- T1 TSE sagittal cervical spine/upper thoracic spine
- T1 TSE sagittal lower thoracic spine/lumbar spine
- STIR sagittal cervical spine/upper thoracic spine
- STIR sagittal lower thoracic spine/lumbar spine
- T1 TSE oblique coronary SIJ*
- STIR oblique coronary SIJ*

* Please cover at least 18 layers and the entire joint, especially ventrally (small pelvis).

Please perform composing of the sagittal sequences.
